# Tape Worms

**Published:** 1874-11

**Authors:** 


Ether for Tape-Worms.—Prof. August Vegel announces its application to tape-worms. The ether is inclosed in a gelatin capsule and swallowed. The ether is vaporized in the stomach and the worm stupefied, it being then easily removed by any of the usual remedies, against which, when awake, the worm offers a strong resistance.—*Cincinnati Lancet and Observer.*

